# Nasopharyngeal carcinoma masquerading as a primary sinonasal mass: a case report

**DOI:** 10.1186/s13256-026-06102-y

**Published:** 2026-05-20

**Authors:** Anurag Lahiri, Niladri Roy

**Affiliations:** Medical College Kolkata, Kolkata, India

**Keywords:** Nasopharyngeal carcinoma, Sinonasal mass, Diagnostic pitfall, Positron emission tomography–computed tomography (PET-CT), Histopathology, Epstein–Barr virus (EBV), Metastasis, Diagnostic Delay, Palliative care

## Abstract

**Background:**

Nasopharyngeal carcinoma (NPC) typically arises from the nasopharyngeal vault and most commonly presents with cervical lymphadenopathy or otologic symptoms. Presentation with a dominant anterior sinonasal mass is exceptionally uncommon and may obscure the true site of origin, leading to diagnostic misclassification as a primary sinonasal malignancy. Cross-sectional imaging, such as computed tomography (CT), may be inconclusive in such cases, necessitating a multimodal approach for accurate diagnosis.

**Case presentation:**

A 54-year-old Indian male presented with progressive unilateral nasal obstruction, recurrent epistaxis, facial swelling, visual impairment, and bilateral hearing loss. Nasal endoscopy revealed a bulky anterior sinonasal mass obstructing visualization of the nasopharynx. CT imaging showed a destructive mass involving the nasal cavity and paranasal sinuses with posterior extension toward the nasopharynx though the primary site remained uncertain. Incisional biopsy revealed a poorly differentiated non-keratinizing carcinoma. Immunohistochemistry (IHC) for p40/p63 and Epstein–Barr virus (EBV)-encoded RNA in situ hybridization confirmed nasopharyngeal carcinoma. Whole-body ^18^F-fluorodeoxyglucose (FDG) positron emission tomography–computed tomography (PET-CT) demonstrated a nasopharyngeal primary with extensive loco-regional invasion, bilateral cervical nodes, and skeletal metastases (cT4bN2cM1). The patient received palliative radiotherapy but deteriorated rapidly and died shortly thereafter.

**Conclusion:**

This case highlights a diagnostic pitfall in which nasopharyngeal carcinoma presents as a predominant anterior sinonasal mass, closely mimicking a primary sinonasal malignancy. Reliance on cross-sectional imaging alone may delay diagnosis. Early biopsy, EBV testing, and multimodal evaluation including PET-CT are essential for accurate tumor localization and staging. Awareness of this atypical presentation may help clinicians avoid diagnostic delay and optimize patient care.

## Background

Nasopharyngeal carcinoma (NPC) is a distinct epithelial malignancy arising from the nasopharyngeal mucosa, most commonly from the fossa of Rosenmüller. It accounts for less than 1% of all malignancies worldwide but demonstrates marked geographic variation, with higher prevalence in southern China and Southeast Asia and a lower yet increasing incidence in regions such as India [1]. The etiology of NPC is multifactorial, with a well-established association with Epstein–Barr virus (EBV) infection, along with environmental exposures, such as nitrosamines and genetic susceptibility [2]. According to the World Health Organization classification, non-keratinizing undifferentiated carcinoma represents the most common histologic subtype and is strongly associated with EBV infection [3].

NPC exhibits aggressive local behavior and a high propensity for early regional nodal and distant metastasis due to its rich lymphatic network and anatomically concealed location near the skull base [4]. Clinically, the most frequent presentation is cervical lymphadenopathy, reported in up to 70–90% of patients, followed by nasal symptoms, such as obstruction and epistaxis, and otologic manifestations related to Eustachian tube dysfunction [5, 6]. Cranial nerve involvement may occur in advanced disease as a result of skull base invasion [7]. Because early symptoms are often mild and nonspecific, diagnosis is frequently delayed, and a significant proportion of patients present with locally advanced or metastatic disease [8].

Although cervical lymphadenopathy and nasopharyngeal symptoms are the most common modes of presentation, nasopharyngeal carcinoma may also present in atypical ways outside its usual clinical pattern. While cranial nerve involvement is classically associated with advanced skull base invasion, rare cases have been reported in which isolated cranial nerve palsy constituted the initial presenting feature [9, 10]. Other unusual presentations described in the literature include multiple cranial nerve involvement mimicking skull base syndromes [11], nonspecific symptoms, such as chronic headache, facial pain, or trismus [12], and, in exceptional cases, distant metastatic manifestations such as vertebral or central nervous system involvement as the first clinical sign [13]. These atypical patterns can obscure the primary site and delay diagnosis.

Local extension of NPC into adjacent structures, including the paranasal sinuses, is a recognized feature of advanced disease. However, presentation with a predominant anterior sinonasal component is uncommon and represents a potential diagnostic challenge. In such scenarios, cross-sectional imaging may demonstrate overlapping features of both nasopharyngeal and sinonasal malignancies, making determination of the primary site difficult and, in some cases, inclining initial diagnostic consideration toward a primary sinonasal tumor [9]. Differentiation from aggressive primary sinonasal malignancies, particularly sinonasal undifferentiated carcinoma (SNUC), may be further complicated when bulky anterior disease limits endoscopic evaluation of the nasopharynx.

In this context, accurate diagnosis often requires a multimodal approach incorporating histopathological examination, EBV-related studies, and advanced imaging to establish the site of origin and extent of disease. We report a case of advanced EBV-associated nasopharyngeal carcinoma presenting with an apparent anterior sinonasal predominance and indeterminate initial imaging findings, illustrating a critical diagnostic pitfall and underscoring the importance of comprehensive clinico-radiologic and pathological correlation.

Reports of NPC presenting with a dominant sinonasal mass remain exceedingly limited, particularly in the Indian population, where delayed diagnosis may further worsen outcomes.

## Case presentation

### Patient background

A 54-year-old Indian male from an urban slum in Kolkata, belonging to a low socioeconomic background, presented to our tertiary-care center with progressively worsening sinonasal and facial symptoms. He had been employed in plastic manufacturing for approximately 20 years and was a chronic smoker. He was a known case of hypertension and type 2 diabetes mellitus, both on regular medication.

### Initial presentation and early disease course

#### September 2024

The patient initially presented to a local clinic with complaints of intermittent right-sided nasal obstruction and purulent nasal discharge. Clinical examination revealed maxillary sinus tenderness, and a provisional diagnosis of chronic sinusitis was made. He was treated conservatively with antibiotics and nasal decongestants, resulting in partial and temporary symptom relief.

**Fig. 1:**
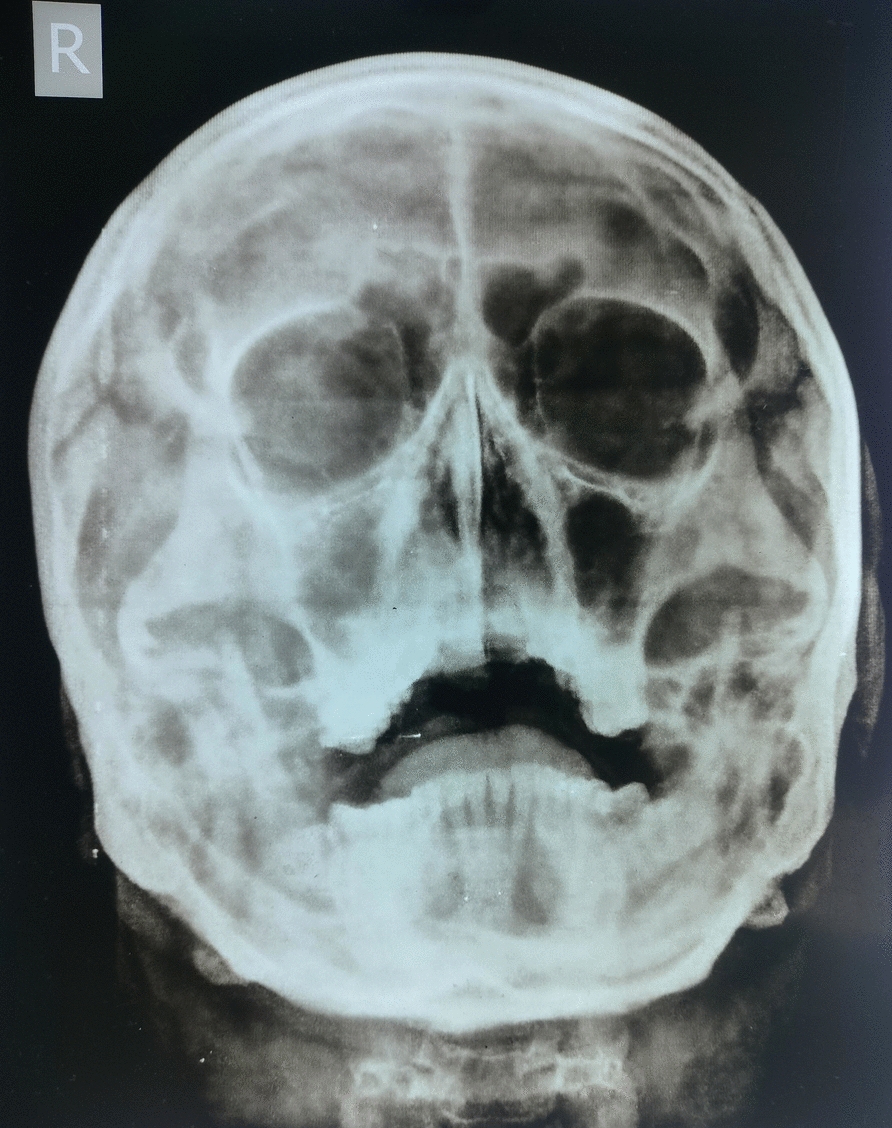
Initial paranasal sinus radiograph showing features suggestive of sinusitis. Water’s view paranasal sinus radiograph shows a midline nasal septum with mild diffuse haziness of the right maxillary sinus, consistent with mucosal thickening. The left maxillary sinus appears relatively clear. No bony erosion, destructive changes, or septal deviation are identified

A paranasal sinus radiograph (Water’s view) was obtained at that time with findings as shown in (Fig. 1).

Over the subsequent four months, the patient made multiple visits to the same local clinic. Despite repeated courses of antibiotics and increasing reliance on topical decongestants, he developed progressive right-sided facial swelling, worsening nasal obstruction, and recurrent epistaxis.

#### December 2024

He was subsequently evaluated at a secondary health facility, where a sinonasal tumor was suspected, and he was referred to our tertiary-care hospital for further evaluation and management.

### Progressive symptoms and clinical examination

#### January 2025

During the four months preceding presentation to our institution, the patient reported recurrent right-sided epistaxis, progressively increasing facial swelling on the right side, and the appearance of a solid intranasal mass extending anteriorly into the right nasal cavity. These symptoms were associated with worsening nasal obstruction, breathing difficulty, and sharp radiating pain over the right ala of the nose.

As the lesion progressed, it expanded medially, compressing the nasal septum and encroaching into the left nasal cavity, resulting in bilateral nasal obstruction, severe septal pain, and marked dyspnea. The patient also complained of disturbed sleep, compensatory mouth breathing, throat dryness, chronic facial pain, and progressive impairment of vision in the right eye.

On examination, there was diffuse right-sided facial swelling with paraesthesia over the right maxillary and zygomatic regions. The mass extended superiorly, compressing the orbit and resulting in loss of vision in the right eye. Anterior rhinoscopy revealed a congested right nasal cavity with mucopurulent discharge and active epistaxis, precluding passage of a nasal endoscope. The left nasal cavity showed septal deviation with areas of cortical erosion along the posterior septum. On palpation, the mass was firm, mildly tender, non-pulsatile, and did not bleed on touch.

**Fig. 2:**
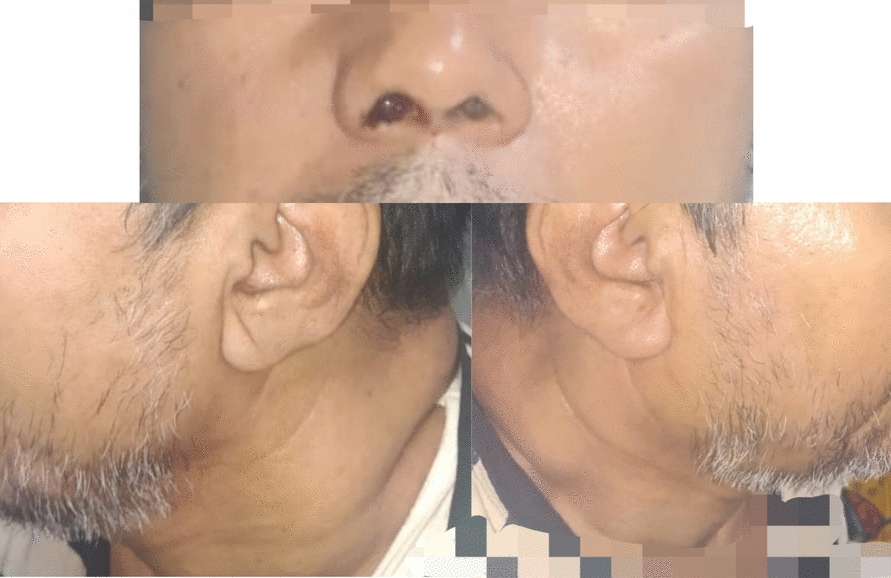
Clinical presentation with facial swelling and cervical lymphadenopathy. Clinical photographs at presentation show right-sided facial fullness and crusted epistaxis at the right nasal vestibule. Lateral views demonstrate multiple enlarged cervical lymph nodes involving the posterior cervical and jugular regions bilaterally, consistent with regional nodal involvement

Multiple cervical lymph nodes were palpable, including the right submandibular, bilateral middle jugular, and left upper posterior triangle nodes. These nodes were firm, non-tender, and reported by the patient to be of recent onset (Fig. 2). The patient also exhibited temporal visual field loss in the left eye and bilateral conductive hearing loss.

**Fig. 3:**
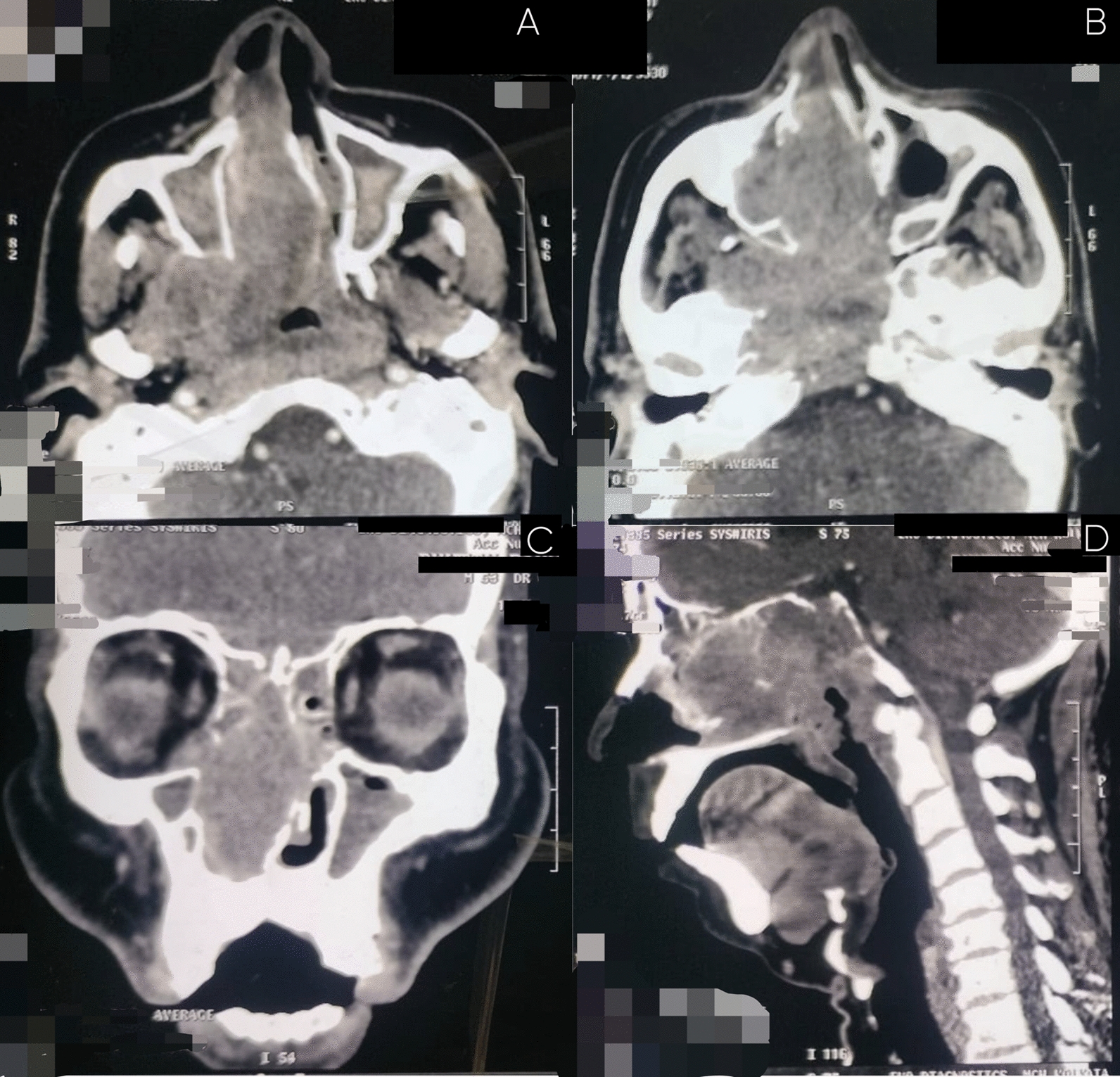
Non-IVCM MDCT of the paranasal sinuses demonstrating extensive loco-regional disease. **A**, **B** Axial sections show an ill-defined, heterogeneously attenuating soft-tissue mass predominantly involving the right nasal cavity, causing anterior nasal airway obstruction, erosion of the nasal septum with deviation toward the left nasal cavity, and posterior extension into the nasopharynx with near-complete obliteration of the nasopharyngeal airway and bilateral involvement of the fossae of Rosenmüller, with loss of adjacent parapharyngeal fat planes. Lateral extension into the right maxillary sinus and superior involvement of the ethmoidal sinus are also noted. **C** Coronal section demonstrates inferior and lateral tumor extension into the right maxillary sinus with erosion of the sinus walls and partial erosion of the orbital floor, indicating advanced local invasion. **D** Sagittal reconstruction reveals posterior skull base involvement with erosion and intracranial extension along the clival region, consistent with aggressive loco-regional spread

A non-intravenous contrast medium (IVCM) multi-detector computed tomography (MDCT) of the paranasal sinuses was performed (Fig. 3), which demonstrated a large locally aggressive soft-tissue mass involving the nasal cavity with posterior extension into the nasopharynx and associated bony destruction. However, the imaging findings were inconclusive in determining the primary site of origin.

#### April 2025

**Fig. 4:**
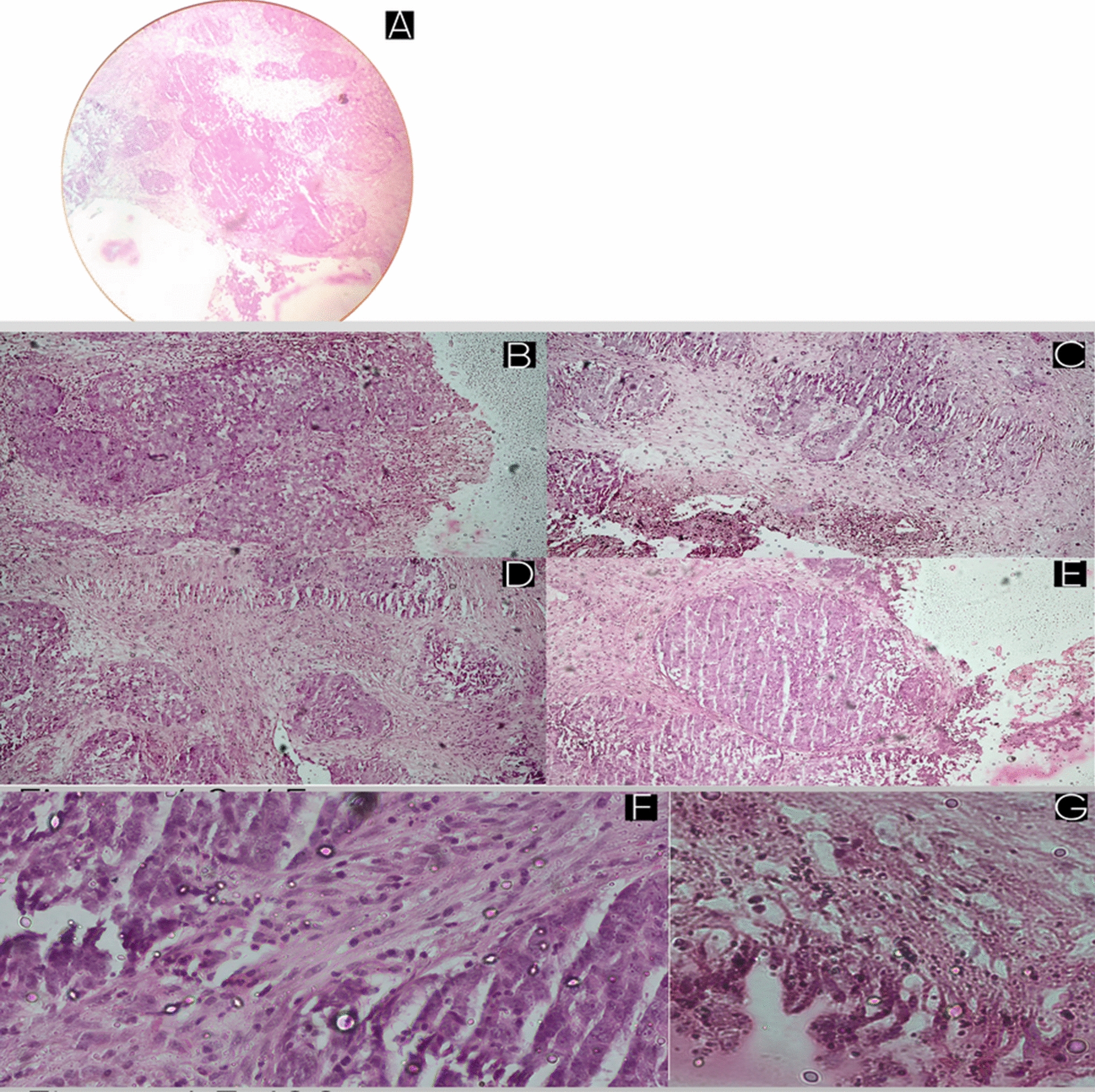
Histopathological features of non-keratinizing undifferentiated nasopharyngeal carcinoma. **A** Low-power view (× 10) showing an infiltrative epithelial neoplasm replacing the normal mucosal architecture and extending into the underlying fibrocollagenous stroma. **B**–**E** Intermediate magnification (× 45) demonstrates varied architectural patterns of the tumor: **B** sheets and syncytial aggregates of malignant epithelial cells; **C** trabecular and cord-like arrangements infiltrating the stroma; **D** irregular infiltrative nests; and **E** a large confluent tumor nest within a dense lymphoplasmacytic stromal background. **F**, **G** High-power views (× 100) reveal tumor cells with indistinct cell borders, vesicular nuclei, prominent nucleoli, marked nuclear pleomorphism, frequent apoptotic bodies, and absence of keratinization, consistent with non-keratinizing undifferentiated carcinoma (nasopharyngeal carcinoma type)

An incisional biopsy obtained from the proliferative mass in the right nasal cavity revealed a poorly differentiated non-keratinizing carcinoma. Histopathological examination demonstrated sheets and syncytial aggregates of malignant epithelial cells with vesicular nuclei, prominent nucleoli, indistinct cell borders, and marked nuclear atypia, embedded within a dense lymphoplasmacytic stroma. There was no evidence of rosette formation, pseudo-glandular architecture, or extensive necrosis. These morphologic features favored a diagnosis of non-keratinizing undifferentiated NPC over SNUC. (Fig. 4).

Immunohistochemistry (IHC) showed tumor cell positivity for p40 and p63, and in situ hybridization for Epstein–Barr virus-encoded RNA (EBER-ISH) was strongly and diffusely positive, confirming EBV-associated NPC.

### Further deterioration and staging

#### May 2025

The patient returned with severe pain and worsening dysphagia. The tumor had progressed to compress the pharyngeal lumen, resulting in absolute dysphagia, generalized weakness, and significant weight loss. A feeding jejunostomy was performed approximately 25 cm distal to the duodenojejunal junction.

#### June 2025

**Fig. 5:**
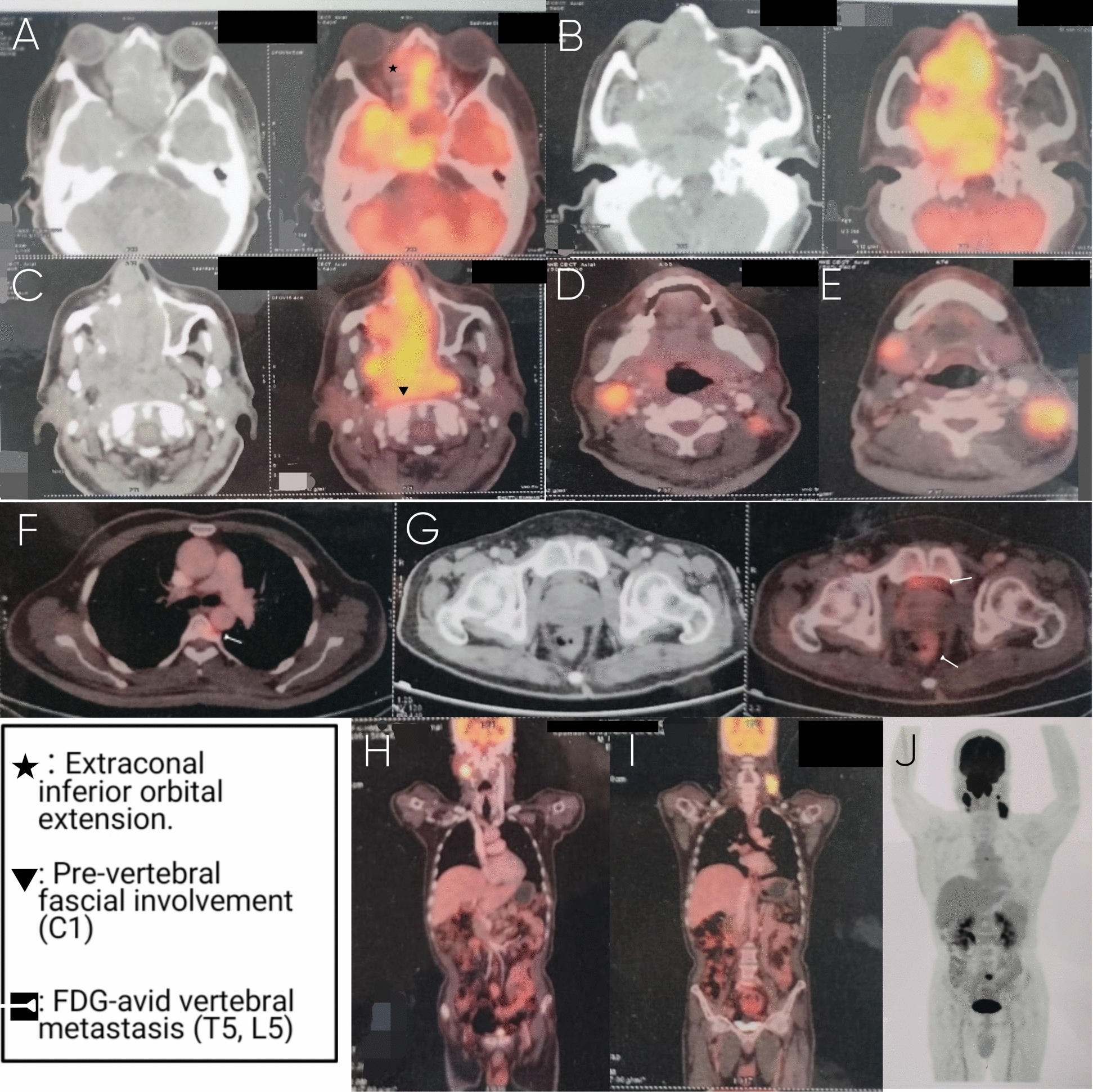
Whole-body ^18^F-FDG PET-CT demonstrating primary nasopharyngeal tumor and metastatic spread. Whole-body ^18^F-FDG PET-CT demonstrates an FDG-avid heterogeneously enhancing soft-tissue mass centered in the nasopharynx, involving both fossae of Rosenmüller, with anterior extension into the right nasal cavity, posterior extension into the prevertebral fascial space at the C1–C2 level, lateral extension into the right maxillary sinus and orbit, superior skull base erosion with intracranial extension, associated cervical lymphadenopathy, and distant skeletal metastases. **A**, **B** Axial CT and fused PET-CT images at the nasopharyngeal level show an intensely FDG-avid mass involving the nasopharynx bilaterally. **C** Axial CT image demonstrates posterior extension of the tumor with loss of the prevertebral fat plane at the C1–C2 level, along with nasal septal erosion and deviation toward the left. **D**, **E** Axial fused PET-CT images demonstrate FDG-avid metastatic cervical lymph nodes. **F** Axial fused PET-CT image of the thorax shows an FDG-avid lytic lesion involving the D5 vertebral body. **G** Axial CT and fused PET-CT images at the pelvic level demonstrate an FDG-avid lytic lesion involving the L5 vertebral body, and anterolisthesis of L5 over S1. **H**, **I** Coronal fused PET-CT images depict extensive metabolically active primary disease with nodal and skeletal metastases. **J** Maximum-intensity projection image demonstrates the overall distribution of FDG-avid disease

A whole-body ^18^F-fluorodeoxyglucose positron emission tomography–computed tomography (^18^F-FDG PET-CT) was performed to assess disease extent and for oncologic staging (Fig. 5). This study demonstrated intense FDG uptake in the pharyngeal region, establishing the nasopharynx as the primary site of origin, despite the tumor’s atypical dominant anterior sinonasal presentation.

A large FDG-avid heterogeneously enhancing soft-tissue mass measuring approximately 9.2 cm (anteroposterior) × 5.4 cm (transverse) × 5.1 cm (craniocaudal), with a Maximum standard uptake (SUVmax) of ~ 11.9, involved the nasopharynx bilaterally, including both fossae of Rosenmüller, with anterior extension into the right nasal cavity. There was associated cortical erosion of the nasal septum with deviation toward the left, along with erosion of the right maxillary antrum, medial orbital wall, and orbital floor, resulting in extension into the extraconal inferior and medial compartments of the right orbit.

Superior extension involved erosion of the clivus, sphenoid bone, and the squamous part of the right temporal bone, with intracranial extension into the sella turcica and the right lateral temporal lobe. Posterior involvement of the prevertebral fascia at the C1–C2 level was noted, without evidence of significant intraspinal extension.

FDG-avid cervical lymphadenopathy was present, involving the right level IB lymph node (SUVmax ~ 5.1, measuring approximately 1.8 × 1.9 cm), bilateral level II lymph nodes (SUVmax up to ~ 10.3, with the largest right level II node measuring ~ 2.5 × 2.2 cm), and the left level V lymph node (SUVmax ~ 8.5, measuring approximately 3.0 × 2.6 cm).

Additionally, FDG-avid lytic skeletal lesions consistent with distant metastases were identified in the D5 vertebral body (SUVmax ~ 4.4) and the L5 vertebral body (SUVmax ~ 10.8). Associated degenerative changes of the lumbar spine with anterolisthesis of L5 over S1 were also noted.

Based on the combined metabolic and anatomic findings, the disease was staged as cT4bN2cM1.

## Treatment and outcome

Given the presence of disseminated skeletal metastases and extensive locoregional disease at presentation, the patient was deemed unsuitable for curative-intent therapy and was planned for palliative treatment. In July 2025, the patient received a single fraction of palliative external beam radiotherapy (8 Gray in 1 fraction) directed to symptomatic spinal sites for pain control.

Systemic chemotherapy was not initiated because of the patient’s poor performance status and rapid clinical deterioration at the time of oncologic assessment, which precluded tolerance of combination therapy. Despite initiation of palliative radiotherapy, the patient’s clinical condition deteriorated rapidly, and he was unable to receive further oncologic treatment. The patient subsequently died due to progressive disease.

The sequence of clinical events from initial presentation to treatment is summarized in Table 1.

**Table 1:** Timeline of the patient’s clinical course

Date	Clinical event
September 2024	Initial presentation to a local clinic with intermittent right-sided nasal obstruction and purulent nasal discharge
September–December 2024	Multiple visits to the same local clinic with persistent symptoms; treated symptomatically
December 2024	Evaluated at a secondary-level healthcare facility; suspected sinonasal tumor and referred to our tertiary-care center
January 2025 (Day 1)	Presentation to our tertiary-care institution with recurrent right-sided epistaxis, progressive right facial swelling, and a solid intranasal mass extending anteriorly
January 2025 (same day)	Clinical examination and radiology (Non-IVCM MDCT)
January 2025 (following week)	Radiologic evaluation reviewed and patient referred for incisional biopsy
April 2025	Incisional biopsy performed; histopathology and EBER-ISH confirmed non-keratinizing undifferentiated nasopharyngeal carcinoma
May 2025	Feeding jejunostomy performed for nutritional support
June 2025	Whole-body PET-CT completed for staging; extensive loco-regional disease and skeletal metastases identified
July 2025	A single fraction of palliative radiotherapy administered
Subsequent course	Clinical deterioration and death shortly thereafter

## Discussion and conclusions

NPC may rarely present with anterior sinonasal predominance, creating significant diagnostic ambiguity and potential delay in definitive management. This case illustrates how reliance on imaging alone may misclassify the tumor origin and emphasizes the decisive role of histopathology, EBV testing, and whole-body PET-CT in establishing the correct diagnosis and stage.

In the present case, marked anterior sinonasal predominance closely simulated a primary sinonasal malignancy both clinically and radiologically. The initial presentation with unilateral nasal obstruction, purulent discharge, facial pain, and epistaxis, coupled with progressive anterior extension that obscured endoscopic visualization of the nasopharynx, effectively masked the primary site of origin. This pattern explains the initial diagnostic trajectory toward a sinonasal primary.

Cross-sectional imaging further contributed to ambiguity. Non-contrast MDCT demonstrated a destructive sinonasal mass with posterior extension but failed to conclusively identify the tumor epicenter. While the overall appearance favored a primary sinonasal carcinoma, posterior extension toward the nasopharyngeal vault, bilateral fossa of Rosenmüller involvement, and early nodal disease raised suspicion for NPC. This highlights the recognized limitation of MDCT in distinguishing anteriorly extending NPC from aggressive sinonasal tumors in locally advanced disease.

Although contrast-enhanced CT or MRI could have provided better anatomical delineation of tumor extent, their use would not necessarily have altered the diagnostic course in this case, as definitive determination of tumor origin requires histopathological confirmation and imaging primarily serves to define local extension and staging rather than establish diagnosis; moreover, contrast administration and prolonged MRI acquisition were limited by poor patient compliance and severe tumor-related pain. In such circumstances, rapid non-contrast multi-detector CT can still provide useful initial information regarding lesion bulk and pattern of spread, facilitating timely biopsy and definitive diagnosis [14].

### Differential diagnoses

From a clinico-radiologic standpoint, a destructive unilateral sinonasal mass with epistaxis and obstruction raises several diagnostic considerations prior to histopathologic confirmation. Nasopharyngeal carcinoma itself may rarely present in ways that mimic other entities, including juvenile nasopharyngeal angiofibroma, as documented in reports describing epistaxis and nasal obstruction with vascular-appearing masses later confirmed as NPC on biopsy [15, 16]. Sinonasal undifferentiated carcinoma represents another primary malignancy with overlapping imaging features, particularly when marked bony destruction and soft-tissue involvement are present [17]. Lymphoproliferative neoplasms, especially extra-nodal NK/T cell lymphoma or other non-Hodgkin lymphomas, should also be considered because extensive sinonasal lymphoid tumors may simulate carcinoma radiologically [18]. Additionally, benign but locally aggressive lesions such as inverted papilloma may present with unilateral obstruction and bone remodeling and can be associated with secondary malignant transformation, further complicating pre-biopsy differentiation [19, 20].

Histopathological evaluation proved decisive. Sinonasal undifferentiated carcinoma (SNUC) represented the principal pathological differential diagnosis because of the aggressive clinical behavior and radiological appearance of the tumor. However, detailed morphologic assessment favored non-keratinizing undifferentiated NPC. The tumor consisted of sheets and syncytial aggregates of malignant epithelial cells with vesicular nuclei, prominent nucleoli, indistinct cell borders, and a dense lymphoplasmacytic stromal infiltrate. These histologic features are characteristic of non-keratinizing undifferentiated NPC as described in standard pathologic classifications, and reflect the typical association of this tumor with an abundant host lymphoid response [3, 21]. In contrast, SNUC is defined by a high-grade undifferentiated carcinoma lacking squamous or glandular differentiation, typically showing marked cytologic pleomorphism, frequent mitoses, extensive necrosis, and absence of a prominent lymphoid background [22, 23]. Furthermore, no rosette formation or pseudo-glandular architecture was identified, arguing against neuroendocrine or other sinonasal primaries.

IHC positivity for p40 and p63 confirmed epithelial differentiation, while strong and diffuse EBER-ISH positivity provided virologic support for EBV-associated NPC, a feature not characteristic of SNUC [3, 21]. Other aggressive sinonasal malignancies, including poorly differentiated squamous cell carcinoma, neuroendocrine carcinoma, and lymphoproliferative neoplasms, were considered less likely in view of the classical morphologic pattern and immuno-phenotype observed. Collectively, these findings indicate that histopathologic examination alone was sufficient to establish the diagnosis of NPC, with IHC serving as confirmatory evidence, thereby underscoring the indispensable role of early biopsy in cases with misleading radiologic appearance.

Whole-body ^18^F-FDG PET-CT played a pivotal role in confirming the nasopharynx as the primary site and defining the full extent of disease. In addition to delineating local invasion and bilateral cervical nodal involvement, PET-CT identified FDG-avid skeletal metastases, establishing advanced-stage disease. The combined metabolic and anatomic assessment resolved uncertainties left by MDCT and enabled accurate staging.

The advanced disease stage at presentation reflected delays at multiple levels. Nonspecific early symptoms delayed initial consultation, while early misclassification as benign sinonasal disease postponed referral and biopsy. Additional procedural and referral-related delays further compounded disease progression. This trajectory underscores the importance of maintaining diagnostic vigilance, pursuing early biopsy in persistent unilateral sinonasal disease, and adopting a multidisciplinary approach in complex head and neck presentations.

### Multilevel contributors to diagnostic delay

In the present case, delayed diagnosis was multifactorial and can be understood using a stepwise delay framework incorporating patient-related and healthcare pathway-related contributors.

The first level of delay occurred at the patient level. The patient belonged to a low socioeconomic background and initially attributed nasal obstruction and epistaxis to benign conditions. Limited health literacy and financial constraints discouraged early presentation to a higher-level facility. Cultural reluctance toward invasive diagnostic procedures such as biopsy further contributed to postponement of definitive evaluation until symptoms became severe and a visible intranasal mass developed.

The second level of delay occurred during the diagnostic workup. Because of poor compliance and difficulty attending repeated hospital visits, investigations were prioritized toward rapid confirmation of malignancy rather than detailed anatomical characterization. Non-IVCM MDCT was performed as an initial imaging modality, followed by referral for tissue diagnosis. However, fear of invasive procedures, logistical barriers and intermittent follow-up resulted in a delay between radiologic evaluation and performance of biopsy, prolonging the time to histopathological confirmation.

The third level of delay occurred between diagnosis and definitive staging and treatment. Advanced imaging and multidisciplinary oncologic care require sustained engagement with the healthcare system. Financial burden, declining performance status, and the anticipated complexity and duration of treatment likely contributed to delayed completion of staging investigations and initiation of therapy. As a result, the disease was ultimately identified at an advanced stage with extensive loco-regional involvement and distant skeletal metastases.

Together, these interacting patient-related and healthcare pathway-related factors explain the prolonged interval between symptom onset and definitive diagnosis. This case highlights how socioeconomic vulnerability, limited health-seeking behavior, and challenges in maintaining continuity of care can substantially influence diagnostic trajectories, even within well-equipped tertiary-care settings.

## Contribution to literature and clinical implications

NPC presenting as a predominant anterior sinonasal mass is exceedingly rare and sparsely described in the literature. Such presentations represent a recognized diagnostic pitfall because extensive anterior tumor extension may obscure the nasopharyngeal epicenter and closely mimic primary sinonasal malignancies both clinically and radiologically, often leading to initial misclassification and delayed diagnosis [5, 24].

The present case further demonstrates how diagnostic ambiguity may persist even with cross-sectional imaging, highlighting the importance of maintaining suspicion for NPC in atypical sinonasal masses. Early tissue biopsy combined with metabolic imaging can be crucial in establishing the true tumor origin when anatomical imaging findings remain inconclusive.

Similarly, Yuszaini *et al.* [17] reported a rare case of NPC masquerading as sinonasal undifferentiated carcinoma, underscoring the potential for misclassification in aggressive tumors with prominent anterior extension, although detailed clinico-radiologic and histopathologic correlation was limited.

By documenting this diagnostic challenge with integrated imaging and histopathologic correlation, the present report expands the spectrum of sinonasal presentations of nasopharyngeal carcinoma and reinforces the importance of early biopsy in atypical sinonasal masses to avoid diagnostic delay.

In conclusion, this case demonstrates that nasopharyngeal carcinoma may rarely present as a dominant anterior sinonasal mass, closely mimicking a primary sinonasal malignancy and thereby constituting an important diagnostic pitfall. It underscores the necessity of maintaining a high index of suspicion for nasopharyngeal origin in destructive unilateral sinonasal lesions and emphasizes the central role of early tissue diagnosis and multidisciplinary evaluation in such presentations.

Beyond its radiologic and pathologic atypia, this case highlights how patient-related factors—including poor compliance, low socioeconomic status, and psychosocial constraints—can substantially influence diagnostic pathways, delay definitive evaluation, and ultimately determine stage at presentation and treatment feasibility. Recognition of these interacting clinical and social determinants is essential to prevent disease upstaging, preserve curative treatment opportunities, and optimize outcomes in patients presenting with atypical patterns of head and neck malignancy.

## Data Availability

All data generated or analyzed during this study are included in this published article. Additional information is available from the corresponding author upon reasonable request.

## Abbreviations

NPC: Nasopharyngeal carcinoma
MDCT: Multi-detector computed tomography
EBV: Epstein–Barr virus
FDG: 18F-fluorodeoxyglucose
IHC: Immunohistochemistry
PET-CT: Positron emission tomography–computed tomography
SNUC: Sinonasal undifferentiated carcinoma
SUVmax: Maximum standardized uptake value
